# Lung infection in orally inoculated SARS-CoV-2 hamsters

**DOI:** 10.1371/journal.pone.0337915

**Published:** 2025-12-04

**Authors:** Evelyne Picard-Meyer, Marine Wasniewski, Franck Boué, Edgar Torres-Maravilla, Vinciane Saint-Criq, Thibaut Larcher, Kevyn Beissat, Elsa Jacouton, Sophie Holowacz, Anne-Judith Waligora-Dupriet, Philippe Langella, Alexandre Servat, Elodie Monchatre-Leroy

**Affiliations:** 1 Nancy Laboratory for Rabies and Wildlife, ANSES, INTERFAS Unit, Malzeville, France; 2 INRAE, Université Paris-Saclay, AgroParisTech, UMR1319 Micalis Institute, Jouy-en-Josas, France; 3 Université Paris-Cité, INSERM, UMRS-1139 FPRM, Paris, France; 4 UMR0703 PAnTher, APEX, INRAE, Oniris VetAgroBio, Nantes, France; 5 VetAgro Sup USC RS2GP, INRAE, VetAgro Sup, Université de Lyon, Marcy l’Etoile, France; 6 PiLeJe Laboratoire, 31-35 Rue de la Fédération, Paris, France; 7 Nancy Laboratory for Rabies and Wildlife, ANSES, SEAR, Malzeville, France; 8 Nancy Laboratory for Rabies and Wildlife, ANSES, Malzeville, France; CEA, FRANCE

## Abstract

The digestive tropism of SARS-CoV-2 due to the presence of the same ACE2 receptor in the digestive system as in the respiratory system raised the question of the role of fecal-oral transmission in the pandemic. The aim of this study was to explore oral infection in hamsters model. SARS-CoV-2 inoculation via the oral and intranasal routes resulted in comparable infection in hamsters. In addition to implications for human health, oral infection could have implications for potential animal reservoirs.

## Introduction

From the very start of the COVID-19 pandemic, symptomatology revealed a SARS-CoV-2 digestive tropism [1]. This digestive and/or respiratory tropism is also found with other coronaviruses, particularly those associated with animal diseases [2], and to a lesser extent those associated with human diseases [3]. This digestive tropism of SARS-CoV-2, due to the presence of the same ACE2 receptor in the digestive system as in the respiratory system, raised the question of the role of fecal-oral transmission in the pandemic [4]. *In vivo* trials have been carried out to further explore this possibility by 2020, in transgenic mice [5], non-human primates [6] and hamsters [7]. These three species inoculated orally or intragastrically, developed clinical signs similar to those caused by intranasal inoculation. As the pandemic unfolded, it became clear that the amount of viral genome in effluents was a fairly reliable indicator of the level of human infection, whatever the variant, even for Omicron, which causes fewer digestive symptoms [8]. It is difficult to estimate the infectivity of the virus in feces [9] and effluents [9,10], but this reinforces the need to better explore oral infection.

The aim of this study was to assess the efficacy in hamster model of oral inoculation with a D614G variant of SARS-CoV-2 which had replaced the original strain tested in one of the rare existing publication [7] on the topic and to compare with the intranasal inoculation by a well-known SARS-CoV-2 variant in hamsters. The implications for the relevance of oral infection in the COVID-19 epidemiology will be discussed in the light of the results such as prevalence of digestive symptoms and infectious excreted viral titer. The potential role of oral infection, along with the emergence of new viral variants, could also increase the likelihood of transmission to other susceptible animal species [11].

## Materials and methods

### Animal experimental design

The experimental protocols complied with the regulation 2010/63/CE of the European Parliament and of the council of 22 September 2010 on the protection of animals used for scientific purposes [12] and as transposed into French law [13]. Experiments were approved by the Anses/ENVA/UPEC ethic committee and authorized by the French Ministry of Research (Apafis n° 33544−2021102114466426 v2).

Thirty-seven-week-old hamsters (*Mesocricetus auratus*, strain RjHan: AURA, Janvier Labs, France) were used. Hamsters were housed in individually ventilated cages with environmental enrichment. Non-infected animals were kept in a separate room from infected animals. Food and water were provided *ad-libitum*. The weight, health condition and activity levels of all animals were monitored and recorded daily (by operators qualified in animal experimentation and welfare) throughout the duration of the experimental procedures (4 or 7 days depending on animal groups). Dyspnea, breathing difficulty, cough and weight loss of more than 15% were used as humane endpoints leading to immediate euthanasia. It must be noted that none of the 30 experimental animal in the present study developed clinical signs set as humane endpoints. All animals were consequently euthanized at the end of the experimental procedure.

For infection, animals were anaesthetized with isoflurane. Twelve anesthetized hamsters were inoculated intranasally (IN) with the dose of 10^5.0^ TCID_50_ (50% tissue culture infectious dose, see paragraph Virus titration) of SARS-CoV-2 (20 µL each nostril). Six hamsters were used as negative control and inoculated IN with 20 µL of PBS in each nostril. Twelve hamsters were infected *per os* (PO) with 10^5.0^ TCID_50_ of SARS-CoV-2 (200 µL for each hamster) without anesthesia by voluntary swallowing of the viral suspension delivered in the mouth with a feeding tube. Six infected hamsters of each group (PO and IN inoculation) were euthanized on 4 days post infection (dpi) and 6 on 7 dpi. Control hamsters were euthanized at 7 dpi. On the day before euthanasia, drinking water was supplemented with Tramadol (0.1 mg/ml) to ensure adequate analgesia for the cardiac blood sampling, which was performed immediately prior to euthanasia. Cardiac blood was collected under deep anesthesia (Isoflurane, Ketamine (150 mg/kg) and Xylazine (10 mg/kg)). Total and terminal blood collection lead to the death of hamsters but a cervical dislocation was carried out as a second method of euthanasia to insure death of animals. After death, all animals were necropsied to collect: feces, lung, nasal turbinates, small and large intestine. Plasma was obtained after blood centrifugation (15 min, 1000 *g*) and stored at −20°C until analysis.

### SARS-CoV-2 virus

The University Hospital of Caen, Normandy, France provided SARS-CoV-2, strain hCoV-19/France/NOR-UCN1/2020 (GISAID reference EPI_ISL_911513). SARS-CoV-2 strain UCN1 was isolated in 2020 during the active epidemic in France. The strain was amplified as described previously and used at passage 2 [14]. The stock P2 was previously verified by sequencing using the Illumina deep sequencing Eurofins Genomics Covid Pipeline v.0.1 (Eurofins Genomics, Ebersberg, Germany). Sequence analysis revealed that the virus had an intact spike cleavage site.

### Virus titration

SARS-CoV-2 titrations from viral inoculum before and after infection and from lung, nasal turbinate samples and digestive samples were performed by TCID50 assay on VERO E6 cells as described in [15].

### RNA extraction, TaqMan real-time RT-qPCR and Digital RT-dPCR

Quantification of virus RNA SARS COV-2 (expressed in number of copies/µl of RNA) was performed by RT-qPCR for lungs and nasal turbinates, as previously described in [14]. Digital RT-dPCR which has the advantage of being less susceptible to inhibitors and provides an absolute target nucleic acid copy number for each sample without the need to run calibration curves, was used for the absolute quantification of SARS COV-2 virus RNA (expressed as copy number/µl of RNA) for jejunum and colon samples.

RNA extraction from nasal turbinates and lung tissues and TaqMan RT-qPCR were performed as previously described in [14]. Briefly, organs (lung (~20 mg) and nasal turbinates (from 16 mg to 74 mg)) were harvested, weighed and placed in a 2 ml Lysing Matrix E FastPrep tubes containing glass beads (MP Biomedicals™), 1 ml of Dulbecco’s modified Eagle medium (DMEM) with 1% antibiotics (Penicillin/Streptomycin (P/S)). The samples were homogenized using a bead beater (MP Biomedicals™ FastPrep-24™ 5G Bead Beating Grinder and Lysis System) at 4 m/s for 10 s, repeated three times with 90 s pauses on melting ice between each cycle. Homogenates were then clarified by centrifugation (2000 g, 10 min, 4°C). Supernatants were collected, aliquoted and stored at −80°C until molecular and virological analyses. TaqMan RT-qPCR was performed with Quantitect Probe RT-PCR kit (Qiagen, Courtaboeuf, France) with E-Sarbeco primer-probes targeting the envelope protein gene (E gene), on the thermocycler Rotor Gene Q MDx (Qiagen, Courtaboeuf, France). Quantification of virus RNA SARS COV-2 was performed as previously described in [14] using a standard curve based on six 10-fold dilutions of a SARS-CoV-2 RNA titrating 3.10^6^ E gene copies/µl of RNA. A threshold setting (Ct) of 0.05 was used as the reference for each RT-qPCR assay.

The jejunum (J) and colon (C) samples were prepared following the same protocol of sample preparation. Briefly, organs (colon (section of 1 cm) and jejunum (section of 1 cm) were harvested, cut into section of 1 cm and the fecal material was removed by pushing. Organs were homogenized in a FastPrep tube (Lysing Matrix E, MP Biomedicals™), containing glass beads and 1 mL of PBS 1x. Tubes were processed on a Fast Prep-24 for three cycles of 30 seconds each at a speed of 4M/s. Homogenates were clarified by centrifugation at 2000 *g*, 4°C for 10 min. Total RNA extraction was done from 350 µL supernatant using the RNeasy^R^ Mini kit, according to the manufacturer’s instructions (QIAGEN, France). RNA was eluted in 50µl RNase free water dispensed directly on the membrane center of the Qiagen RNeasy spin mini column, then stored at <−76°C.

RT-dPCR was performed on the QIAcuity One 5-plex platform (Qiagen, France) using the same model primer-probe targeting the E gene, described in [14]. The reaction mixture (40 µL) consisted of RNA sample (5 µL), 4x OneStep advanced probe master mix (10 µL), 100x OneStep Advanced RT mix (0.4 µL), 10 µM forward (F1) and reverse (R2) primers (3.2 µL each), 10 µM probe (P1) (0.8 µL) and RNAse free water (17.4 µL).

The reaction mixture was transferred into Qiacuity 26k 24-well Nanoplates (Qiagen, France) for partitioning into 26000 partitions using the Qiagen Standard Priming Profile, followed by PCR reactions on each of the partition. The following protocols were carried out for PCR amplification: 50°C for 40 min for reverse transcription, 95°C for 2 min and 45 cycles at 95°C for 5 s and 60°C for 30 s, and 3) a final imaging step in the FAM channel (300 ms – Gain 4).

QIAcuity Suite Software version 2.2.0.26 (Qiagen, France) was used for data analysis and quantities were reported as copies per microliter of reaction mixture, then expressed in copies/µL of RNA.

The threshold was positioned between the positive and negative partition at 39.2.

Negative (RNase free water) and positive (SARS-COV-2 RNA) controls were included in the experiments.

The limit of blank was set at 1 copy/µL.

### Serological analyses

The detection of anti-SARS-CoV-2 antibodies in the plasma samples was performed by microsphere immunoassays (MIAs) as described in [16]. Three recombinant SARS-CoV-2 antigens (nucleoprotein, spike subunit 1 RBD and spike trimeric (The Native Antigen Company)) were used to capture specific plasma antibodies, whereas BSA (Bovine Serum Albumin) was used as a control antigen in the assay. Plasma samples were previously diluted 1:400 in assay buffer (PBS-1% BSA-0.05% Tween 20). Measurements were performed using a Bio-Plex 200 instrument (Luminex technology). At least 100 events were read for each bead set and binding events were displayed as median fluorescence intensities (MFI). Relative fluorescence intensities (RFI) were calculated for each sample by dividing the MFI signal measured for the antigen-coated microsphere sets by the MFI signal obtained for the control microsphere set, to account for non-specific binding of antibodies to beads. Specific seropositivity cut-off values for each antigen were set at three standard deviations above the mean RFI of negative samples. The seroconversion was established only when antibodies were detected for the three antigens [15].

### Histological analyses

Representative samples from the jejunum, colon and lungs were fixed in formalin and paraffin embedded. A minimum of two and a maximum of three consecutive 5 µm-thick sections, per organ and per animal, were mounted on a glass slide, stained with routine hematoxylin-eosin-saffron and were scanned using Panoramic SCAN 3DHISTECH (Zeiss objective × 20). All analyses were performed on scanned sections using Qu-Path software (v. 0.4.2) [16]. Samples were analyzed by a senior veterinary pathologist and all lesions were recorded and semi-quantitatively assessed.

In colon and jejunum samples, incidences of macroscopic vasoplegia and inflammatory cell infiltration were recorded. In lungs, the following nine parameters were scored after observation of all whole sections, with a 4-level scale (from 0 to 3): (i) Absence (0) or presence (1) of intra-bronchial debris or pus leading to partial (2) or total (3) occlusion of the bronchial lumen; (ii) Absence (0) or presence of alveolar emphysema with over-inflated (1), destroyed (2) or collapsed (3) air sacs; (iii) Normal vessels (0) or vasculitis with intramural leukocytoclasis (1), media necrosis (2) or transmural infiltration (3); (iv) No (0) or focal (1), coalescing focal (2) or extensive (3) alveolar edema; (v) No (0) or focal (1), coalescing focal (2) or extensive (3) hemorrhages; (vi) no (0), mild (1), marked (2) or severe (3) alveolar wall thickening; (vii) No (0) or focal (1), coalescing focal (2) or extensive (3) of alveolar lumen occlusion (consolidation); (viii) No (0) or focal (1), coalescing focal (2) or extensive (3) type II pneumocyte hyperplasia and (ix) No (0) or focal (1), coalescing focal (2) or extensive (3) inflammatory cell infiltration of lung parenchyma. A total composite score was further calculated (sum of all individual scores, max. cumulative score = 27).

This descriptive analysis was completed by a quantitative assessment of pneumonia. Using pixel classifier tool from QuPath software, a machine training was performed by the pathologist to differentiate lung parenchyma infiltrated with inflammatory cells from normal. This training was done on a panel of representative pictures and then applied to one whole section of lung per animal. The percentage area of pneumonia was further calculated as the ratio between area recognized as infiltrated and the total surface area of the lung parenchyma.

### Proteolytic activity

Fecal protease activity was determined photometrically by using azocasein as a proteolytic substrate [17,18]. Each fecal sample (50 mg) was mixed with 1 mL of reaction buffer (0.5% W/V NaHCO3, pH 8.3) and homogenized. The homogenate was then centrifuged at 1800 *g* for 10 minutes at 4°C. The resulting supernatant from the fecal homogenate was incubated with 100 μL of reaction buffer and 100 μL of Azocasein solution (0.5% W/V azocasein in reaction buffer, Sigma Aldrich) at 40°C for 20 minutes. The reaction was terminated by adding 100 μL of 10% V/V trichloroacetic acid (Sigma Aldrich). Following a second centrifugation at 1800 g for 10 minutes at 4°C, the absorbance of the clear supernatants was measured at 450 nm using a microplate reader.

### Statistical analyses

As data did not meet the normality assumptions, non-parametric tests were used in order to compare: 1) PO and IN inoculation for each time point and 2) the negative group with the other two groups (PO and IN).

The Kruskal-Wallis test and its appropriate post-hoc test for multiple comparison, Dunn’s test were used for statistical analysis of RNA titers (Fig 2 and Fig 3), histological read out (Fig 6) and proteolytic activity (Fig 7) (Prism GraphPad V9 and V10.2.3).

**Fig 1:**
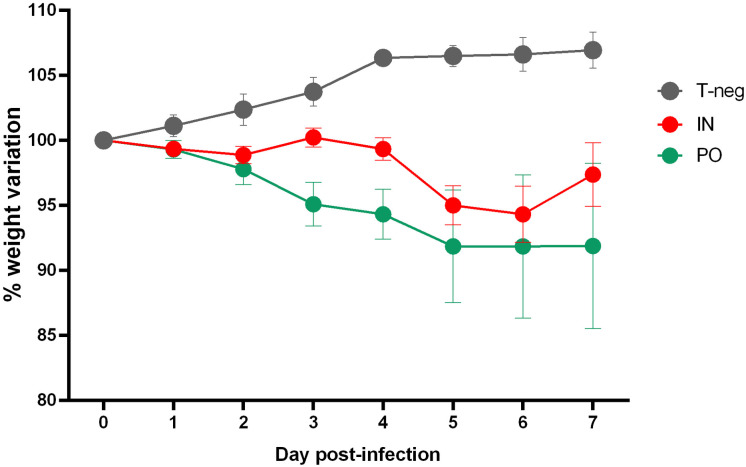
Follow-up on the weight of hamsters from 0 dpi to 7 dpi. Weight changes (%, mean ± SD) from 0 to 7 dpi after intranasal (IN in red) or *per os* (PO in green) inoculation with SARS-CoV-2 or IN inoculation with PBS (T-neg in grey). For IN and PO inoculated group, n = 12 from dpi 0 to dpi 4 and n = 6 from dpi 4 to dpi 7. For T-neg group, n = 6 from dpi 0 to dpi 7.

**Fig 2:**
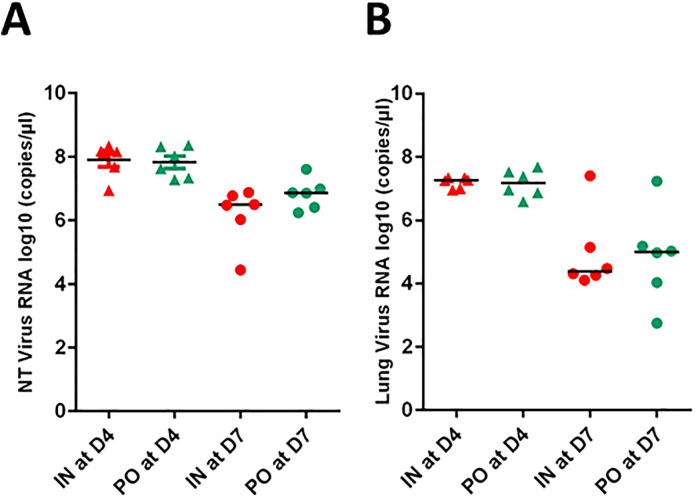
Viral RNA loads in the respiratory system (nasal turbinates and lungs). Viral RNA loads in the nasal turbinates (NT) **(A)** and in the lungs **(B)** in the two groups of hamsters inoculated with SARS-CoV-2. Bars represent medians. Viral RNA loads are marked by triangles at 4dpi (D4) and by circles at 7dpi (D7). Animals inoculated by the intranasal route are in red and in green by *per os* route (IN: intranasal, PO: *per os*).

**Fig 3:**
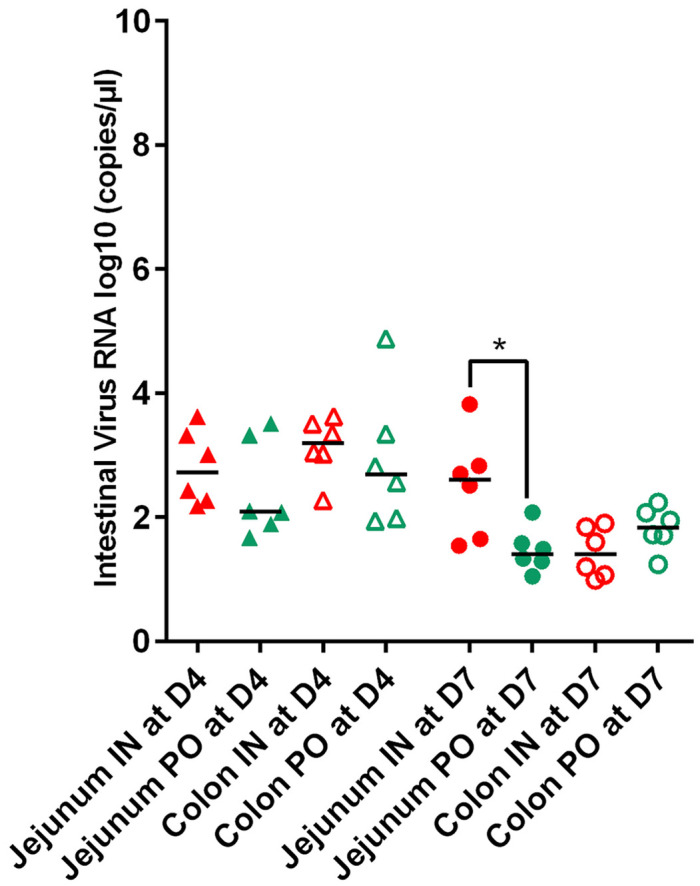
Viral RNA loads in the colon and jejunum. Viral RNA loads in the colon (empty shape) and jejunum (full shape) in the two groups of hamsters inoculated with SARS-CoV-2. Bars represent medians. Viral RNA loads are marked by triangles at 4dpi (D4) and by circles at 7dpi (D7). Animals inoculated by the intranasal route are in red and in green by *per os* route (IN: intranasal, PO: *per os*). Significant (*p < 0.05).

**Fig 4:**
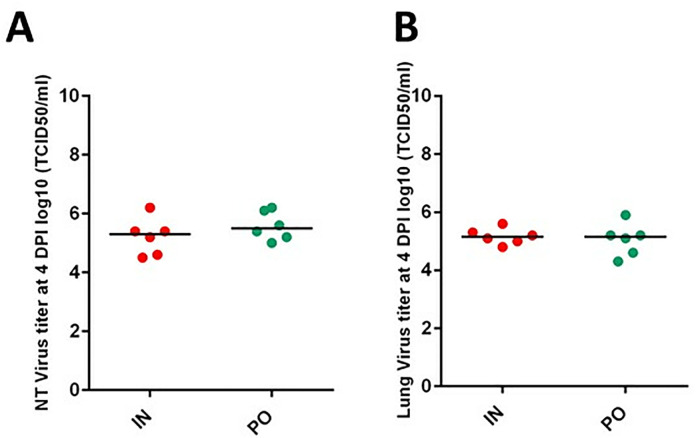
Viral titers in the nasal turbinates and lungs. Viral titers at 4 dpi in the nasal turbinates (NT) **(A)** and lungs **(B)** of the two groups of hamsters inoculated with SARS-CoV-2 (IN: intranasal inoculation in red, PO: *per os* in green). Bars represent medians. At 7 dpi, no infectious viral particle was detected in the lungs of inoculated hamsters. However, some infectious particles were detected in the nasal turbinates of one hamster belonging to the IN group but at a low titer (≤ 10^2.1^ TCID50/mL, value not shown). At 4 dpi and at 7 dpi, no infectious particle was detected in the digestive system.

**Fig 5:**
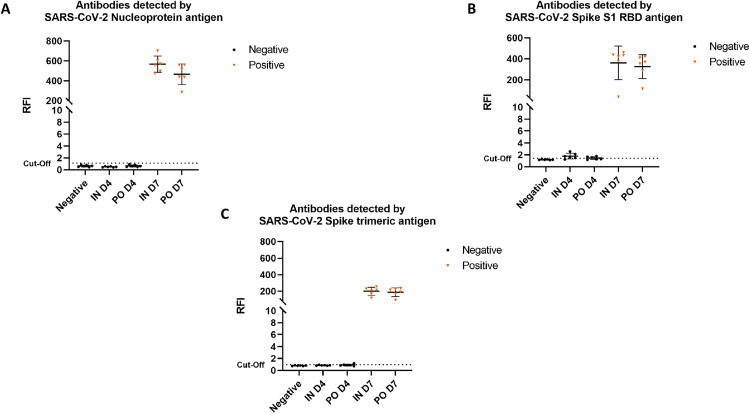
Detection of anti-SARS-CoV-2 antibodies at 4 and 7 dpi. Serological evaluation of anti-SARS-CoV-2 antibodies at 4 and 7 dpi (D4 and D7) in negative controls and hamsters inoculated with SARS-CoV-2 intranasally (IN) or *per os* (PO). **(A)** Anti-N antibody levels. **(B)** Anti-S1 RBD antibody levels. **(C)** Anti-Trimeric Spike antibody levels. SARS-CoV-2 specific antibody levels were assessed using microsphere immunoassays (MIAs) and expressed as relative fluorescence intensities (RFI) to control antigen. The negative group was used to determine the cut-off (mean + 3 standard deviations). For A, B and C, lines are means ± 95% confidence intervals. Seroconversion was established only when antibodies were detected for three antigens.

**Fig 6:**
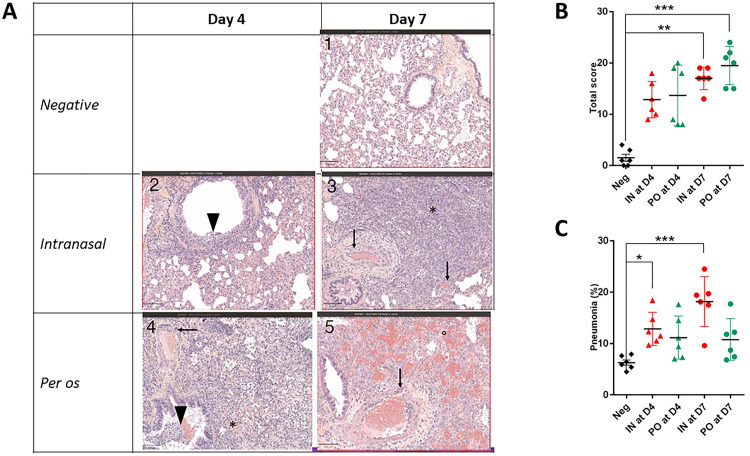
Histopathology of lung tissues of inoculated hamsters. **(A)** Representative images from hematoxylin-eosin-saffron (HES) staining of lung tissue of hamsters inoculated intranasally with SARS-CoV-2 show broncho-interstitial pneumonia at 4 dpi (▼) and suppurative bronchitis and proliferative acute injury (*) with vasculitis (↓) at 7 dpi. *Per os* inoculation induced proliferative acute lung injury (*) and vasculitis (↓) at 4 dpi and exudative acute lung injury (°) with massive hemorrhages and vasculitis (↓) compared to negative controls (PBS). **(B)** Total lung histological score for the negative control group (PBS inoculated hamsters), IN group (hamsters inoculated intranasally with SARS-CoV-2) and PO group (hamsters inoculated *per os* with SARS-CoV-2) at 4 (D4) and 7 (D7) dpi. **(C)** Pneumonia-associated consolidation (%) for the negative control group (PBS inoculated hamsters), IN group (hamsters inoculated intranasally with SARS-CoV-2) and PO group (hamsters inoculated *per os* with SARS-CoV-2) at 4 (D4) and 7 (D7) dpi. Significant (*p < 0.05; ** p < 0.01; *** p < 0.001).

**Fig 7:**
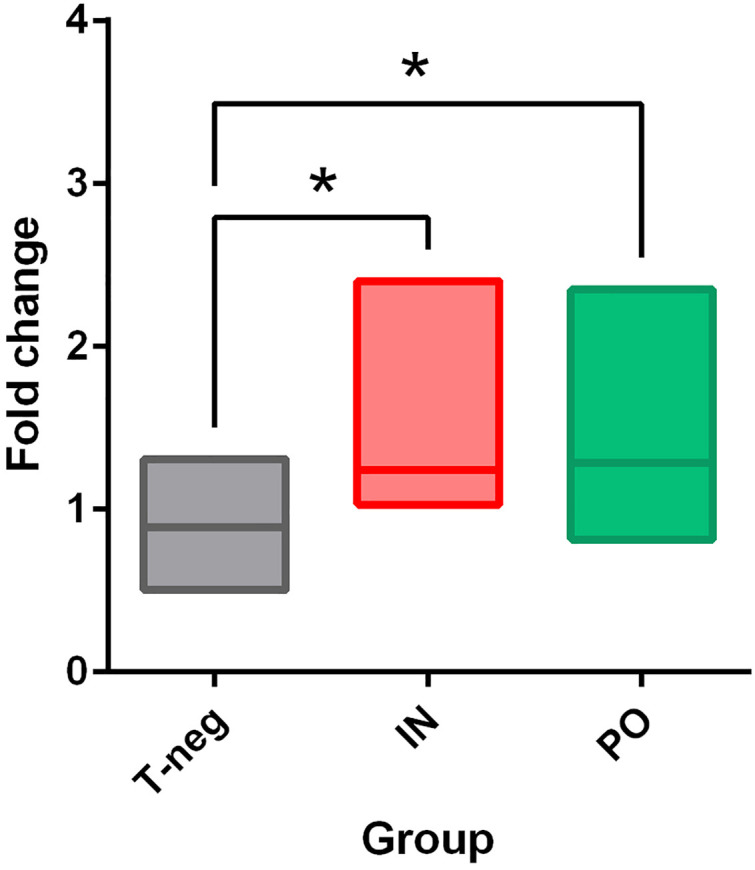
Intestinal proteolytic activities at 7 dpi in hamsters. Proteolytic activity changes at 7 dpi after intranasal (IN in red) or *per os* (PO in green) inoculation with SARS-CoV-2 or IN inoculation with PBS (T-neg in grey). Significant (*p < 0.05).

Statistical analysis of the two groups of infectious viral titers in Fig 4 was done by Wilcoxon and its appropriate post-hoc test, Mann-Whitney test (package stat in R, version 4.1.2).

## Results

### No difference in clinical observations between PO and IN hamsters

No death and no clinical endpoint were reached during the entire observation period. The body weight of infected hamsters decreased during the 7 days of experimentation whereas it increased in non-infected hamsters during the same period. The maximum average weight loss observed was 8% in PO infected hamsters on dpi 7. The maximum average weight loss observed was 5% in IN infected hamsters on dpi 6. Over the same period, non-infected controls showed an average weight gain of 7% (Fig 1).

### Detection of viral genome mainly in respiratory system of hamsters from both groups

Viral RNA was detected in nasal turbinates at 4 and 7 dpi in both infected groups. The number of log10 RNA copies/µl of samples varied from 4.44 to 8.36 with larger variations at 7 dpi (Fig 2). Viral RNA copies were not statistically different (p > 0.9) between the IN and PO groups in the lungs and nasal turbinates at both time points. No viral RNA was detected in the negative control group.

Finally, viral RNA was detected at 4 dpi and 7 dpi in the jejunum and colon of both groups of hamsters inoculated with SARS-CoV-2. Number of log10 RNA copies/µL of samples varied from 1 to 4.8 (Fig 3). RNA titers showed high variability in comparison with respiratory system. No trend was observed in relation to the part of digestive system (colon *versus* jejunum). Viral RNA titers were not statistically different (p > 0.06) between IN and PO groups whatever the tissue (colon or jejunum) or the DPI concerned (4dpi or 7 dpi).

### No detection of infectious virus in digestive system of hamsters from both groups

At 4 dpi, infectious viral particles were detected in respiratory system of IN and PO groups with titers from 4.30 to 5.90 log10 TCID50/mL in lungs and with titers from 4.5 to 6.2 log10 TCID50/mL in nasal turbinates (Fig 4). Virus titers were not statistically different (p > 0.3) between IN and PO groups.

### Seroconversion of hamsters from both groups

The anti-SARS-CoV-2 antibodies were detected with the three recombinant SARS-CoV-2 antigens (nucleoprotein, spike subunit 1 (S1) RBD and spike trimeric) at 7 dpi indicating a seroconversion in all infected animals, whereas no antibody was detected at 4 dpi (Fig 5). There was no difference regarding the anti-SARS-CoV-2 antibodies response between the IN and the PO groups.

### Differences in histopathology at 7 dpi between PO and IN hamsters

Hamsters inoculated intranasally and *per os* showed significant lung lesions (Fig 6). More specifically, IN infection caused broncho-interstitial pneumonia at 4 dpi and suppurative bronchitis and proliferative acute injury with vasculitis at 7 dpi (Fig 6A). PO infection induced proliferative acute lung injury and vasculitis at 4 dpi and exudative acute lung injury with massive haemorrhages and vasculitis at 7 dpi (Fig 6A). No lung lesion was observed in the negative control group. The total lung histological score showed that IN- and PO-infections cause similar lesions in the lungs (Fig 6B). At 4 dpi, infection led to a non-significant increase in lesions (Fig 6B) in comparison with the negative control group, whatever the route of infection. At 7 dpi, both modes of infection (IN and PO) resulted in lesions with significantly higher scores compared to the negative control group. The IN group showed a significantly higher pneumonia-associated consolidation than the PO group at 7 dpi (Fig 6C).

In the jejunum samples, no vasoplegia or inflammation was observed in the negative control group (Table 1). The incidence of vasoplegia increased from 4 to 7 dpi in the IN group. Vasoplegia was detected in all PO-infected hamsters at 4 dpi and 7 dpi. Only samples of PO-infected hamsters presented inflammation.

**Table 1:** Incidence of vasoplegia and inflammation in the jejunum of sampled hamsters.

Group	Vasoplegia	Inflammation	Total number of sampled hamsters
Negative control group	0	0	6
SARS-COV-2 IN at 4 dpi	2	0	6
SARS-COV-2 PO at 4 dpi	5	4	5
SARS-COV-2 IN at 7 dpi	6	0	6
SARS-COV-2 PO at 7 dpi	5	2	5

In the colon samples, minimal mononuclear cell infiltration was sometimes observed, mainly at 7 dpi in the IN group and at 4 and 7 dpi in the PO group. This was a very unspecific lesion and the relation with the experimental infection is uncertain.

### Disturbance of the proteolytic activity of the digestive system during viral infection

Intestinal proteolytic activity significantly increased in infected animals regardless of the inoculation route compared to the negative control group (Fig 7).

The data supporting this article have been included as part of the Supplementary Information, detailed in S1 Table.

## Discussion

In the present study, the SARS-CoV-2 experimental challenge of two groups of hamsters (either by the oral (PO) or intranasal (IN) route) was similarly successful at inducing weight loss in both groups, seroconversion within 7 days and viral infection (viral RNA load and titration) of the lungs, upper respiratory tract and gut tissues, as well as proteolytic activity changes as seen in COVID-19 patients [19,20]. However slightly more severe inflammatory levels were seen in the gut of the PO group and pneumonia-associated consolidation seemed higher in the IN group. In both groups, the viral RNA levels were lower and more variable in the gut than in the upper respiratory tract and the lungs, but still significant. This study therefore confirms that oral inoculation of SARS-CoV-2 can induce infection in the pulmonary tract in hamsters as also seen by Lee et al. [7], and in non-human primates [6]. This is not true for human ACE2 mice [5]. Although Lee et al. [7] showed that oral inoculation of SARS-CoV-2 can induce a milder pneumonia in hamsters, and lower viral load in the lungs than IN inoculation, we saw limited impact of the challenge routes in our study, possibly because the variant delivered to hamsters carried mutations in Spike (D614G and F367V), in ORF8 (S84L) and in ORF1ab (T5020I). D614G and F367V mutations are known to increase viral affinity with ACE2 [21,22] and the S84L mutation is associated with more severe lesions [23].

A more severe inflammation (pneumonia-associated consolidation) was seen in the lungs of the IN group, and vasoplegia and inflammation of the jejunum were clearer in the PO group. The inoculation routes may therefore explain some differences seen in clinical symptoms in COVID-19 patients, in particular those presenting digestive signs [24–26]: they may be caused by the indirect effects of circulating inflammatory cytokines and other mediators or from the direct cytopathogenic effects of the virus infecting the digestive system from the main respiratory infection site *via* the sputum [26]. Rezzoug et al. [27] highlighted a correlation between the levels of respiratory viral load and the viral RNA in faeces, suggesting that the respiratory infection may expand, at a lower level, to the digestive tract. Our results are consistent with this hypothesis, since both oral and respiratory inoculation routes resulted in a respiratory infection, but also in a milder digestive infection. How the virus delivered orally may contaminate the pulmonary track in our study remains to be explained, but one likely hypothesis is that a cross infection occurred when the infectious bolus was swallowed and allowed the virus to spread along the respiratory epithelium of the larynx or pharynx to the lungs and the nasal volutes. This spreading pathway has already been suggested for influenza viruses that are transmitted orally [28] but also cause respiratory symptoms with or without digestive symptoms [29].

Although the IN route remains the main cause of severe clinical and histopathologic signs in patients in a dose dependent manner [7], our study raises the question of the importance of oral infection in COVID-19 human cases, at least for some mutations as D614G that may have become dominant over others through this enhanced spreading capacity. Indeed, a significant amount of virus being excreted into the environment *via* droplets [30] and surviving for hours to days on surfaces [31,32], could remain infectious on hands, food or any other object placed in the mouth and result in new oral infection [4]. However one should remain cautious in extrapolating conclusions from our study regarding the impact of infectious oral doses, since those used in our study were known to be infectious for hamsters [33–35] but the average infectious dose for humans is still not fully understood and probably depends on many factors such as the viral variant, environmental conditions, and individual immunity [34]. It would therefore be interesting to quantify the dose effects of different SARS-CoV-2 variants delivered orally to non-human primates.

In addition to impacting human health, oral contamination causing an infection similar to that of intranasal contamination could also impact the epidemiology of SARS-CoV-2 infections in animals. Indeed, infection by the oral route from contaminated fomites could facilitate transmission from humans to animals. The high infection rate (30–40%) reported in free-ranging white-tailed deers (*Odocoileus virginianus*) in the U.S. [36] may result from indirect oral infection, since direct contact between humans and deer are very rare. Similarly, if SARS-CoV-2, widely detected at the molecular level in wastewater is infectious [9], the risk of a reservoir among rodents living in sewers cannot be ignored.

## Conclusion

Our study suggests that the importance of oral infections should be considered in COVID-19 epidemiology, impacting treatment and prevention not only in humans, but also in animals.

## Supporting information

S1 Table Raw data shown in Figures 1–7. S.Fig 1, Bodyweight of SARS COV-2 infected hamsters shown in Fig 1; S.Fig 2, Detection of SARS COV-2 RNA at D4 and D7 shown in Fig 2; S.Fig 3, PCR raw data in the jejunum and the colon shown in Fig 3; S.Fig 4, Raw data of viral titers in the nasal turbinates and lungs shown in Fig 4; S.Fig 5, Detection of anti-SARS-CoV-2 antibodies at 4 and 7 dpi shown in Fig 5; S.Fig 6, Histopathology of lung tissues of inoculated hamsters shown in Fig 6; S.Fig 7, Intestinal proteolytic activity changes at 7 dpi in hamsters shown in Fig 7.
(XLSX)
